# Predictive models of radiographic progression and pain progression in patients with knee osteoarthritis: data from the FNIH OA biomarkers consortium project

**DOI:** 10.1186/s13075-024-03346-1

**Published:** 2024-05-30

**Authors:** Xiaoyu Li, Chunpu Li, Peng Zhang

**Affiliations:** 1 Department of Orthopedics, Qilu Hospital of Shandong University (Qingdao), Shandong University, Shandong, 266000 China; 2 Key Laboratory of Qingdao in Medicine and Engineering, Qilu Hospital of Shandong University (Qingdao), Shandong University, Shandong, 266000 China

**Keywords:** Knee osteoarthritis, Predictive model, Radiographic progression, Pain progression

## Abstract

**Objectives:**

The progression of knee osteoarthritis (OA) can be defined as either radiographic progression or pain progression. This study aimed to construct models to predict radiographic progression and pain progression in patients with knee OA.

**Methods:**

We retrieved data from the FNIH OA Biomarkers Consortium project, a nested case-control study. A total of 600 subjects with mild to moderate OA (Kellgren-Lawrence grade of 1, 2, or 3) in one target knee were enrolled. The patients were classified as radiographic progressors (*n* = 297), non-radiographic progressors (*n* = 303), pain progressors (*n* = 297), or non-pain progressors (*n* = 303) according to the change in the minimum joint space width of the medial compartment and the WOMAC pain score during the follow-up period of 24–48 months. Initially, 376 variables concerning demographics, clinical questionnaires, imaging measurements, and biochemical markers were included. We developed predictive models based on multivariate logistic regression analysis and visualized the models with nomograms. We also tested whether adding changes in predictors from baseline to 24 months would improve the predictive efficacy of the models.

**Results:**

The predictive models of radiographic progression and pain progression consisted of 8 and 10 variables, respectively, with area under curve (AUC) values of 0.77 and 0.76, respectively. Incorporating the change in the WOMAC pain score from baseline to 24 months into the pain progression predictive model significantly improved the predictive effectiveness (AUC = 0.86).

**Conclusions:**

We identified risk factors for imaging progression and pain progression in patients with knee OA over a 2- to 4-year period, and provided effective predictive models, which could help identify patients at high risk of progression.

**Supplementary Information:**

The online version contains supplementary material available at 10.1186/s13075-024-03346-1.

## Introduction

Osteoarthritis (OA) is the most common form of arthritis in the elderly population, and knee OA is a major cause of physical disability [1]. With the increasing burden of OA on individuals and society, appropriate and timely treatment is essential to prevent disease progression. Knee OA progresses in different ways, as most patients experience gradual worsening over decades, and some experience rapid deterioration. The heterogeneity in knee OA progression presents a challenge in clinical decision-making and the design of clinical trials. Early identification of patients who are prone to rapid progression is crucial for the development of preventative measures and disease-modifying treatments.

Predictive models of knee OA progression can facilitate the development of suitable management strategies for individual OA patients and the selection of patients for clinical trials. Structural progression does not always coincide with symptomatic progression. Pain sensitization may occur in patients reporting high levels of pain without evidence of moderate-to-severe pathological changes [2]. Treatment of knee OA can be planned according to radiological findings and functional status.

Using data from the FNIH OA Biomarkers Consortium Project, this study constructed and evaluated predictive models of radiographic parameters and pain progression for knee OA based on logistic regression analysis. We selected the candidate variables from the broad range of variables collected from the database. The candidate variables included not only demographic data, clinical and imaging measurements but also biochemical markers. We sought to construct cross-validated models that use baseline data and changes at 24 months to predict disease progression between 24 and 48 months. To make the predictive models easy to use, we visualized them with nomograms. Our models may contribute to a deeper understanding of the pathophysiology of OA and assist in treatment decisions and the design of clinical trials.

## Methods

### Subjects

**Fig. 1:**
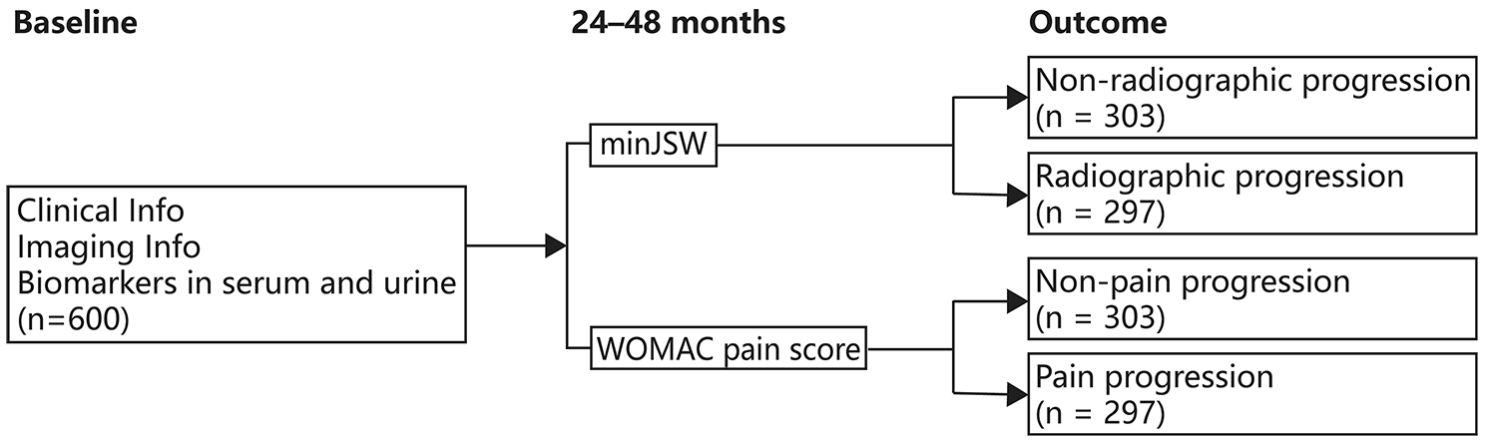
Study design. *Note* minJSW: minimum joint space width; WOMAC: Western Ontario and McMaster Universities Arthritis Index

The data used in this study were from the FNIH OA Biomarkers Consortium project, a nested case-control study of knee OA progression biomarkers, within the Osteoarthritis Initiative (OAI) [3]. The OAI was measured in a longitudinal cohort (4,796 men and women aged 45–79 years) at the onset and/or progression of knee OA. Imaging of both knees of participants, blood and urine specimens, and clinical data from both knees of the participants were obtained at baseline and annual follow-up. The FNIH project selected 600 subjects with one index knee per subject from the OAI to identify potential biomarkers of knee OA progression. Eligible subjects had at least one knee with a Kellgren-Lawrence grade (KLG) of 1, 2, or 3 and were classified into one of four groups based on the outcome in the index knee over the 24–48 month follow-up (Fig. 1). The four knee outcomes were (1) both radiographic and pain progression (*n* = 194); (2) radiographic progression but not pain progression (*n* = 103); (3) pain progression but not radiographic progression (*n* = 103); and (4) neither radiographic nor pain progression (*n* = 200). For covariate balance among the groups, index knees selected for the four groups were frequency matched for 15 strata of the KLG (1 or 2 or 3) by body mass index (BMI; kg/m^2^) category (< 25; 25 to < 27.5; 27.5 to < 30; 30 to < 35; ≥35).

### Disease progression definition

Radiographic progression was defined as a decrease of ≥ 0.7 mm in the minimum joint space width (minJSW) of the medial femorotibial compartment (MFTC) from baseline to 24, 36, or 48 months [4]. Pain progression was defined as a persistent increase of ≥ 9 points on a 0100 normalized score of the Western Ontario and McMaster Universities Arthritis Index (WOMAC) pain score from baseline to 24, 36, or 48 months [5]. Pain persistence required a pain increase of ≥ 9 points at two or more points from the 24- to 60-month pain assessment. Knee pain was assessed using the WOMAC version LK 3.1. The 5-point Likert scale version of the WOMAC questions will be used, modified from the original format to ask about the right and left knee separately during the past 7 days.

### Exclusion criteria

Patients who underwent total knee or hip replacement or had metal implants in the bone from baseline through 24 months were excluded due to potential effects on biochemical markers. Knees that were unable to meet the criteria for radiographic or pain progression due to ceiling effects at baseline (minimum medial joint space width < 1.0 mm and/or WOMAC pain > 91 on a 0–100 scale) were excluded. In addition, knees with predominantly lateral compartment joint space narrowing at baseline or during follow-up were excluded.

### Variable selection

We started by considering all variables from the baseline dataset. We removed metadata variables (e.g., dates, IDs) and variables with missing values. This resulted in 376 variables (Supplementary Table 1). We transformed the right and left knee variables into target and nontarget knee variables. Categorical variables with levels less than 30 in the reference group were reclassified as binary categorical variables based on median values. We tested differences in patient characteristics between the radiographic progression and non-radiographic progression groups and between the pain progression and non-pain progression groups. We also assessed the significance of each variable by univariate logistic regression analysis to investigate the risk factors for radiographic progression and pain progression. Variables associated with radiographic progression or pain progression at a significant level were candidates for corresponding multivariate analysis.

### Building predictive models

We performed a multivariate logistic analysis based on the previously mentioned potential risk factors. Ten events per variable is a widely advocated minimal criterion for sample size considerations in logistic regression analysis [6]. As there were 297 patients in both the radiographic progression group and the pain progression group, the maximum number of variables included in the regression analysis was 29. There were no two variables in the model with a Spearman correlation coefficient greater than 0.5. The variance inflation factor (VIF) was used to analyze the collinearity of various factors in the logistic regression analysis. A VIF > 10 was considered indicative of multicollinearity. To ensure that the predictive models are generalizable to new data, we used 10-fold cross-validation for performance evaluation and model selection. The Akaike information criterion (AIC) was used to determine the variables for the multivariate logistic regression models. We generated nomograms based on multivariate analysis. The area under the curve (AUC) based on the receiver operating characteristic (ROC) curve was measured to test the discrimination of the nomograms, and the AUCs were compared using DeLong’s test. Furthermore, a calibration curve was used to evaluate the calibration of the nomogram. A Hosmer‒Lemeshow test was performed to support the calibration. The internal validation was completed by bootstrapping of 1,000 repeated samplings to reduce the bias of excessive fitting. Decision curve analysis (DCA) was performed to determine the clinical validity of the nomogram by measuring the net benefits at different threshold probabilities.

### Statistical analysis

Data analysis was conducted using R (R 4.1.2). Continuous variables are presented as the means ± standard deviations (SDs) or medians (1st quartile, 3rd quartile). Normally distributed data were tested using Student’s t-test, and nonnormally distributed data were analyzed by the Mann‒Whitney U test. Categorical variables are expressed as numbers (percentages) (N (%)) and were analyzed with chi-square tests and Fisher’s exact tests. All tests were two-sided, and the significance level was set as *p* < 0.05.

## Results

### Baseline characteristics

The age of the subjects ranged from 55 to 69 years at baseline. There were significant differences in age, sex, race, and KLG between the non-radiographic progression group and the radiographic progression group at baseline (Table 1). The WOMAC pain score was significantly lower in the pain progression group than in the non-pain progression group. The analysis results of all baseline variables are shown in Supplementary Tables 2–5.

**Table 1:** Baseline characteristics of the patients

Variable	Total (n = 600)	Non-radiographic progression (n = 303)	Radiographic progression (n = 297)	P value	Non-pain progression (n = 303)	Pain progression (n = 297)	P value
Age, yr	61 (54, 69)	60 (53, 68)	62 (55, 69)	0.017	62 (54.5, 69)	60 (54, 68)	0.144
b, n (%)				0.002			0.77
Male	247 (41)	106 (35)	141 (47)		127 (42)	120 (40)	
Female	353 (59)	197 (65)	156 (53)		176 (58)	177 (60)	
Race, n (%)				0.037			0.258
Other	125 (21)	74 (24)	51 (17)		57 (19)	68 (23)	
White or Caucasian	475 (79)	229 (76)	246 (83)		246 (81)	229 (77)	
Kellgren-Lawrence grade				0.002			0.511
1	75 (12)	37 (12)	38 (13)		38 (13)	37 (12)	
2	306 (51)	175 (58)	131 (44)		161 (53)	145 (49)	
3	219 (36)	91 (30)	128 (43)		104 (34)	115 (39)	
Medial minimum joint space width, mm	3.83 (2.93, 4.67)	3.89 (3.1, 4.59)	3.67 (2.82, 4.73)	0.16	3.88 (2.98, 4.65)	3.74 (2.87, 4.67)	0.696
WOMAC Pain Score	1 (0, 4)	1 (0, 4)	1 (0, 4)	0.513	1 (0, 5)	1 (0, 3)	0.019

WOMAC: Western Ontario and McMaster Universities Arthritis Index

WOMAC pain scale: Likert version, range 0–20

### Development and validation of the predictive model

**Fig. 2:**
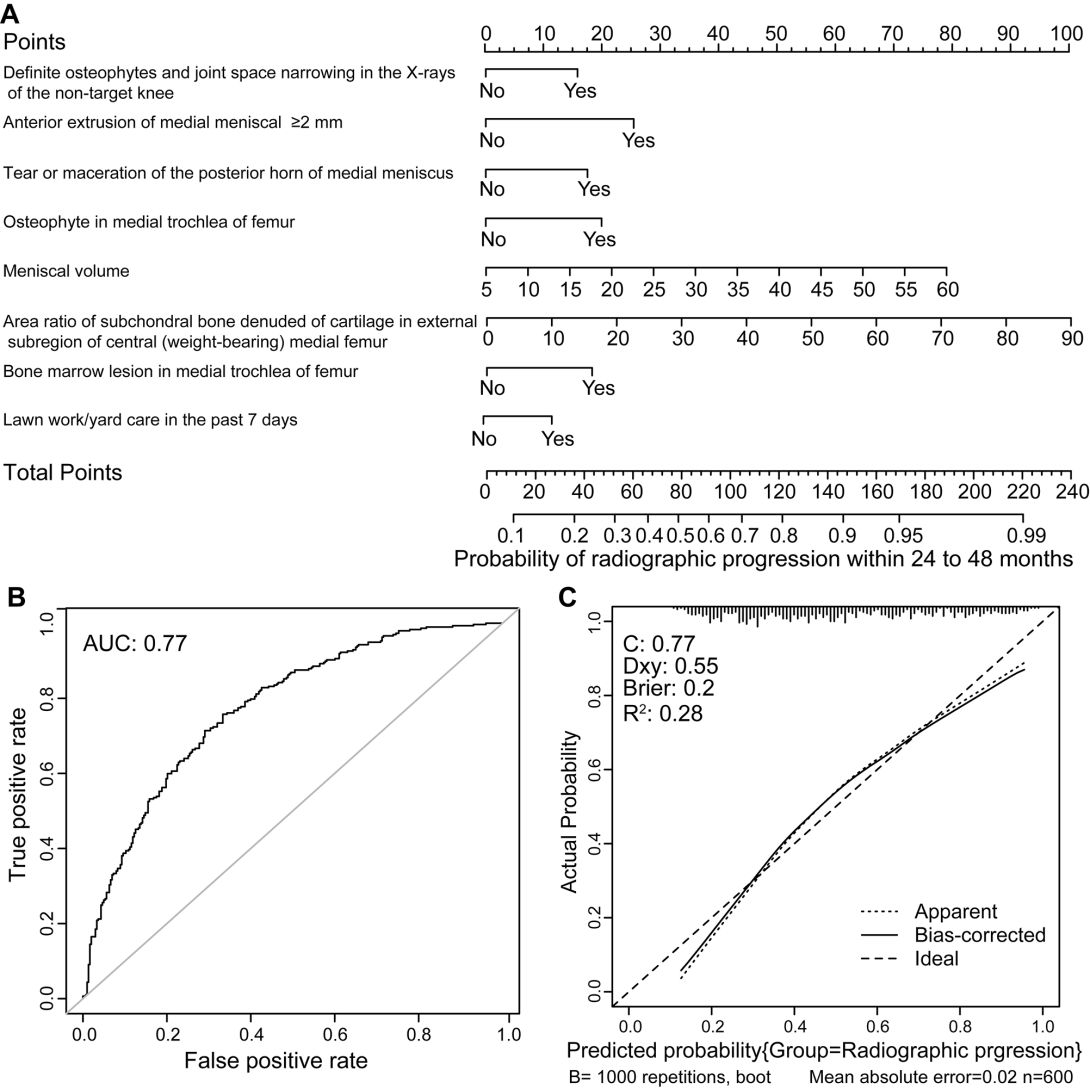
Nomogram for predicting radiographic progression at baseline and its predictive performance. (*A*) Nomogram to estimate the risk of radiographic progression. To use the nomogram, find the position of each variable on the corresponding axis, draw a line to the points axis for the number of points, add the points from all of the variables, and draw a line from the total points axis to determine the probabilities of radiographic progression at the lower line of the nomogram. (*B*) Receiver operator characteristic curve for the nomogram predicting radiographic progression. AUC: area under curve. (*C*) Calibration curves of the nomogram predicting radiographic progression

According to the univariate analysis, 115 and 75 candidate variables were selected for radiographic progression modeling and pain progression modeling, respectively. Subsequently, we performed multivariate logistic regression analysis using 10-fold cross-validation. The combination of variables with the lowest AIC values was used to develop the predictive models. The combination of variables with the lowest AIC in the radiographic progression predictive model included 28 variables (Supplementary Table 6), 8 of which were significant in the multivariate logistic regression model (Table 2). The 8 variables were medial meniscal extrusion anteriorly ≥ 2 mm, meniscal volume, osteophytes in the medial trochlea of the femur, the area ratio of subchondral bone denuded of cartilage in the external subregion of the central (weight-bearing) medial femur, tear or maceration of the posterior horn of the medial meniscus, bone marrow lesion (BML) in the medial trochlea of the femur, definite osteophytes and joint space narrowing in the X-rays of the nontarget knee, and lawn work/yard care in the past 7 days. The nomogram predicting radiographic progression based on the 8 variables is shown in Fig. 2, with an AUC of 0.77, and the calibration plot shows good agreement between the prediction and actual incidence of radiographic progression.

**Table 2:** The results of multivariate logistic analysis of the selected variables for the nomogram predicting radiographic progression at baseline

Variable	Label	Coefficient	OR (95% CI)	P value
V00MMXMA	MOAKS: medial meniscal extrusion ≥ 2 mm - anteriorly	8.21E-01	2.27 (1.53–3.40)	5.54E-05
V00MedialMeniscus	Meniscal volume (100 mm^3) - medial	4.65E-02	1.05 (1.02–1.27)	8.13E-05
V00MOSFMA	MOAKS: osteophyte - femur medial anterior (trochlear)	6.43E-01	1.90 (1.31–2.78)	7.83E-04
V00EBMFPD	% area of subchondral bone denuded of cartilage - central medial femur (external) [%]	3.60E-02	1.04 (1.02–1.06)	1.68E-03
V00MMTMP	MOAKS: tear or maceration of medial meniscus - posterior horn	5.65E-01	1.76 (1.21–2.56)	3.19E-03
V00MBMSFMA	MOAKS: bone marrow lesions - femur medial anterior (trochlear)	5.84E-01	1.79 (1.18–2.73)	6.15E-03
P01L(R)XRKOA2	Non-target knee baseline x-ray: definite osteophytes and joint space narrowing	5.10E-01	1.66 (1.13–2.46)	1.07E-02
V00HOUACT4	Household activities: lawn work/yard care, past 7 days	3.80E-01	1.46 (1.01–2.12)	4.42E-02

OR: odds ratio; CI: confidence interval; MOAKS: MRI Osteoarthritis Knee Score

**Fig. 3:**
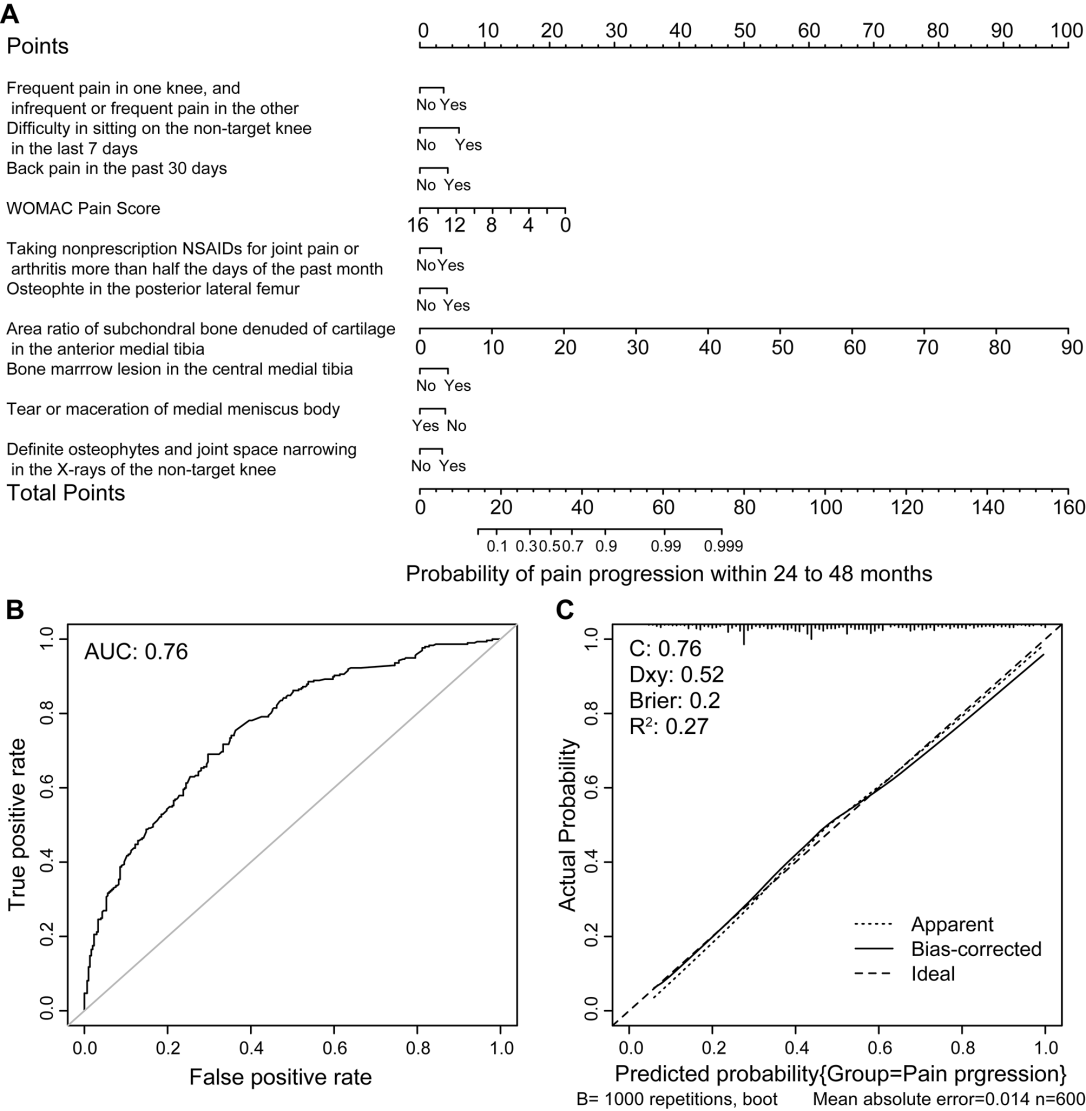
Nomogram for predicting pain progression at baseline and its predictive performance. (**A**) Nomogram to estimate the risk of pain progression. WOMAC: Western Ontario and McMaster Universities Arthritis Index; WOMAC pain scale: Likert version, range 0–20; NSAID: non-steroidal anti-inflammatory drugs. (**B**) Receiver operator characteristic curve for the nomogram predicting pain progression. AUC: area under curve. (**C**) Calibration curves of the nomogram predicting pain progression. *Note* The dashed diagonal line represents the ideal prediction of the ideal model and the solid line represents the performance of the nomogram; the areas of closer approach indicate better prediction

Similarly, the combination of variables with the lowest AIC in the pain progression predictive model had 19 variables (Supplementary Table 7), 10 of which were significant in the multivariate logistic regression model (Table 3). The 10 variables were frequent pain in one knee and infrequent or frequent pain in the other, difficulty sitting on the nontarget knee in the last 7 days, back pain in the past 30 days, WOMAC pain score, nonprescription nonsteroidal anti-inflammatory drugs (NSAIDs) for joint pain or arthritis for more than half of the past month, osteophytes in the posterior lateral femur, the area ratio of subchondral bone denuded of cartilage in the anterior medial tibia, BML in the central medial tibia, tear or maceration of the medial meniscus body, and definite osteophytes and joint space narrowing in the X-rays of the nontarget knee. The nomogram for predicting pain progression based on the 10 variables is shown in Fig. 3, with an AUC of 0.76, and the calibration curve showed that the nomogram prediction results were consistent with the actual results.

**Table 3:** The results of multivariate logistic analysis of the selected variables for the nomogram predicting pain progression at baseline

Variable	Label	Coefficient	OR (95% CI)	P value
V00WOMKP	WOMAC Pain Score	-2.30E-01	0.79 (0.74–0.85)	4.98E-10
V00DIL(R)KN14	Non-target knee difficulty: sitting, last 7 days	9.88E-01	2.68 (1.67–4.39)	6.01E-05
P01BP30	Back pain, past 30 days	7.04E-01	2.02 (1.39–2.96)	2.46E-04
V00MOSFLP	MOAKS: osteophyte - femur lateral posterior	6.84E-01	1.98 (1.35–2.93)	5.64E-04
V00MMTMB	MOAKS: tear or maceration of medial meniscus - body	-6.42E-01	0.53 (0.35–0.79)	2.26E-03
V00MBMSTMC	MOAKS: bone marrrow lesion - tibia medial central	7.10E-01	2.03 (1.29–3.23)	2.32E-03
P01KSX	Frequent pain in one knee, and infrequent or frequent pain in the other	6.00E-01	1.82 (1.22–2.74)	3.67E-03
P01L(R)XRKOA2	Non-target knee baseline x-ray: definite osteophytes and joint space narrowing	5.64E-01	1.76 (1.19–2.61)	5.04E-03
V00AMTPD	% area of subchondral bone denuded of cartilage - medial tibia (anterior) [%]	1.82E-01	1.20 (1.08–1.43)	9.78E-03
V00NSAIDS	Taking nonprescription NSAIDs (e.g., Aspirin, Ibuprofen…) for joint pain or arthritis more than half the days of the month, past 30 days	5.40E-01	1.72 (1.09–2.73)	2.12E-02

OR: odds ratio; CI: confidence interval; WOMAC: Western Ontario and McMaster Universities Arthritis Index; WOMAC pain scale: Likert version, range 0–20; MOAKS: MRI Osteoarthritis Knee Score; NSAID: non-steroidal anti-inflammatory drugs

### Improvement and comparison of models

**Fig. 4:**
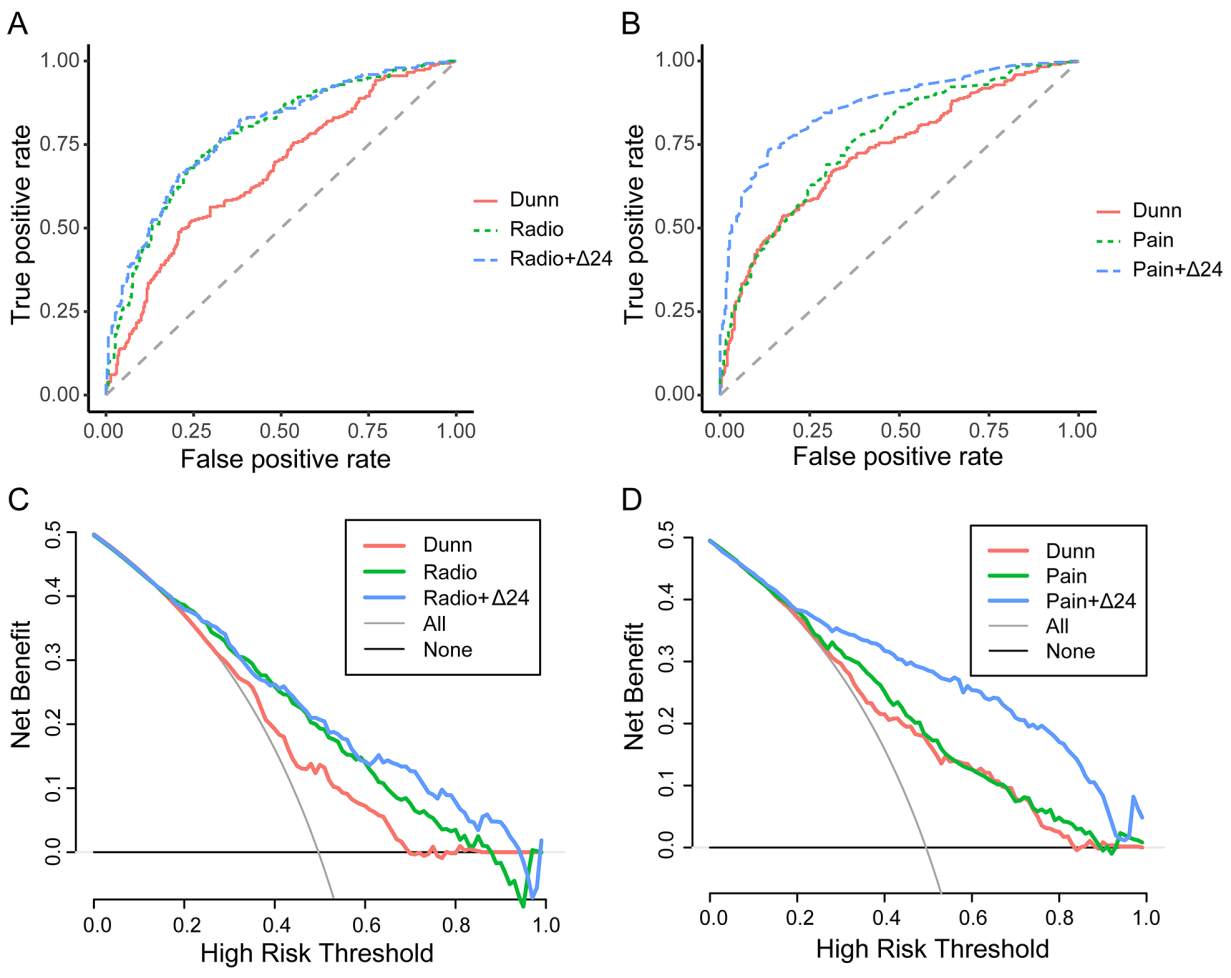
Receiver operator characteristic (ROC) curves and decision curve analysis (DCA) for nomograms. (**A**) ROC curves of nomograms predicting radiographic progression. (**B**) ROC curves of nomograms predicting pain progression. (**C**) DCA of nomograms predicting radiographic progression. (**D**) DCA of nomograms predicting pain progression. *Note* Dunn indicated nomograms based on the variables suggested by Dunn et al.; Radio and Pain indicated nomograms based on baseline characteristics; and Radio + Δ24 and Pain + Δ24 indicated nomograms based on baseline characteristics and the changes during the 24-month follow-up period

To improve the prediction capability, we added the changes in numerical variables from baseline to 24 months to the predictive model. According to the predictive model of radiographic progression, the changes in the area ratio of subchondral bone denuded of cartilage in the external subregion of the central (weight-bearing) medial femur were significant according to the multivariate regression model (Radio + Δ24), with an AUC of 0.78 (Table 4; Fig. 4A). For the predictive model of pain progression, the change in the WOMAC pain score was significant according to the multivariate regression model (Pain + Δ24), with an AUC of 0.86 (Table 5; Fig. 4B).

**Table 4:** Multivariate logistic analysis of baseline variables and changes over 24 months for predicting radiographic progression

Variable	Label	Coefficient	OR (95% CI)	P value
V00MedialMeniscus	Meniscal volume (100 mm^3) - medial	5.29E-02	1.05 (1.03–1.08)	8.21E-06
ΔEBMFPD	Changes in area ratio of subchondral bone denuded of cartilage from baseline to 24 months - central medial femur (external) [%]	9.93E-02	1.10 (1.06–1.17)	5.55E-05
V00MMXMA	MOAKS: medial meniscal extrusion ≥ 2 mm - anteriorly	6.45E-01	1.91 (1.27–2.87)	1.98E-03
V00MOSFMA	MOAKS: osteophyte - femur medial anterior (trochlear)	5.55E-01	1.74 (1.19–2.56)	4.34E-03
V00MMTMP	MOAKS: tear or maceration of medial meniscus - posterior horn	5.47E-01	1.73 (1.18–2.54)	5.38E-03
V00MBMSFMA	MOAKS: bone marrow lesions - femur medial anterior (trochlear)	5.89E-01	1.80 (1.18–2.76)	6.59E-03
V00EBMFPD	% area of subchondral bone denuded of cartilage - central medial femur (external) [%]	2.99E-02	1.03 (1.01–1.06)	9.34E-03
P01OXRKOA2	Non-target knee baseline x-ray: definite osteophytes and joint space narrowing	4.84E-01	1.62 (1.08–2.43)	1.86E-02

MOAKS: MRI Osteoarthritis Knee Score

**Table 5:** Multivariate logistic analysis of baseline variables and changes over 24 months for predicting pain progression

Variable	Label	Coefficient	OR (95% CI)	P value
ΔWOMKP	Changes in WOMAC pain score from baseline to 24 months	6.06E-01	1.83 (1.64–2.07)	1.44E-24
V00DIL(R)KN14	Non-target knee difficulty: sitting, last 7 days	8.52E-01	2.34 (1.40–3.96)	1.24E-03
V00MOSFLP	MOAKS: osteophyte - femur lateral posterior	6.65E-01	1.94 (1.27–2.99)	2.33E-03
V00AMTPD	% area of subchondral bone denuded of cartilage - medial tibia (anterior) [%]	2.05E-01	1.23 (1.09–1.46)	4.52E-03
V00MBMSTMC	MOAKS: bone marrrow lesion - tibia medial central	5.44E-01	1.72 (1.07–2.80)	2.64E-02
P01BP30	Back pain, past 30 days	4.49E-01	1.57 (1.03–2.39)	3.58E-02
V00NSAIDS	Used nonprescription NSAIDs (e.g., Aspirin, Ibuprofen…) for joint pain or arthritis more than half the days of the month, past 30 days	5.52E-01	1.74 (1.04–2.92)	3.58E-02

OR: odds ratio; CI: confidence interval; WOMAC: Western Ontario and McMaster Universities Arthritis Index; WOMAC pain scale: Likert version, range 0–20; MOAKS: MRI Osteoarthritis Knee Score; NSAID: non-steroidal anti-inflammatory drugs

We also compared our models with the model constructed by Dunn et al. [7]. Dunn et al. developed a Cox model to predict end-stage knee OA using the following 9 variables: KLG for the target knee, Knee Injury and Osteoarthritis Outcome Score (KOOS) quality of life score, Osteoarthritis Research Society International (OARSI) medial joint space narrowing of the target knee, degree of flexion contracture/hypertension in the target knee, pain severity (0–10) in the target knee in the past 30 days, WOMAC disability score of the target knee, symptomatic OA status of the target knee, WOMAC pain score of the target knee, and KLG score for the nontarget knee. The AUCs of the radiographic progression predictive model and the pain progression predictive model based on the 9 variables were 0.67 and 0.73, respectively (Fig. 4A, B).

The AUC of the Radio + Δ24 model was not significantly different from that of the Radio model but was significantly greater than that of the Dunn model (*p* < 0.001). DCA curves showed that the net benefit of the Radio + Δ24 model was slightly better than that of the Radio model across a narrow range of thresholds (0.5–0.9) (Fig. 4C). The AUC of the Pain + Δ24 model was significantly greater than that of the Pain and Dunn models. The DCA showed that the benefit of the Pain + Δ24 model was greater than that of the Pain and Dunn models at a threshold range of approximately 0.3 to 0.9 (Fig. 4D).

## Discussion

In this study, we created models to predict radiographic progression and pain progression in patients with knee OA based on clinical questionnaires, imaging measurements, and molecular biomarkers. To obtain the greatest power of discrimination and calibration, we first screened all the variables that we could initially retrieve from the OAI database. Then, we performed multivariate logistic regression to construct the predictive models and nomograms. The final predictive models for radiographic progression and pain progression included 8 and 10 predictors, respectively. Notably, 6 of the 8 predictors in the predictive model of radiographic progression and 4 of the 10 predictors in the predictive model of pain progression were dependent on MRI. In the predictive model of pain progression, four predictors were pain-related queries. Osteophyte and joint space narrowing in the nontarget knee is a common predictor in both the predictive model of radiographic progression and the predictive model of pain progression. In addition, by using the ROC curves and DCA, we found that incorporating the changes in the WOMAC pain score from baseline to 24 months promoted the predictive performance of the predictive model of pain progression.

Three MRI-based predictors were associated with the meniscus in the predictive model of radiographic progression. Medial meniscus extrusion has been reported to be related to the incidence of radiographic knee OA (defined as KLG ≥ 2) in middle-aged overweight and obese women over 30 months follow-up [8]. An MRI study showed substantial medial meniscus extrusion (> 3 mm) correlated with severe meniscal degeneration, extensive tear, complex tear, large radial tear, and tear involving the meniscal root [9]. Our findings suggest that anterior extrusion of the medial meniscus greater than or equal to 2 mm may be an early marker for the development of OA.

Although the meniscus plays a critical protective role for the knee, we found that excessive meniscal volume increases the chance of radiographic progression. In our study, the median (1st quartile, 3rd quartile) baseline medial meniscus volume for the non-radiographic progression group and radiographic progression group were 2245.73 (1822.51, 2916.81) mm^3^ and 2617.91 (2096.58, 3247.54) mm^3^, respectively (Supplementary Table 2). Our results are consistent with Xu et al. [10]. They found that that a larger baseline volume of the medial meniscus and the decrease of meniscal volume over time were associated with the incidence of radiographic knee OA after 30 months of follow-up in overweight and obese women. Recently they found that varus alignment, higher BMI, more meniscus pathologies, meniscus extrusion, fewer cartilage lesions, lower physical activity level, higher quadriceps muscle strength, and higher age were associated with greater meniscus volume in women aged between 45 and 60 years without radiographic signs of knee OA or clinical diagnosis of OA [11]. The large meniscus volume may reflect a compensatory change in OA and the modifiable factors related to meniscus volume (e.g., varus alignment, BMI, physical activity level, and quadriceps muscle strength) may guide the prevention of knee OA.

An anatomical study found that radial tearing in the posterior horn of the medial meniscus was a risk factor for cartilage degeneration [12]. An MRI study found that posterior root tear of the medial meniscus was associated with anteriorly medial meniscal degeneration, medial and posterior extrusion of the medial meniscus, and greater medial tibial posterior slope in medial-type OA knees with a KLG of 2 or less [13]. Our findings suggest that tear or maceration of the posterior horn of the medial meniscus can independently predict the radiographic progression of knee OA in the presence of medial meniscal extrusion and medial volume.

The other three MRI-based predictors in the predictive model of radiographic progression were measurements of the medial femur. A cohort study found that patellofemoral cartilage damage was associated with developing osteophytes in the patellofemoral compartment in middle-aged subjects without radiographic knee osteoarthritis during 72 months of follow-up [14]. A retrospective radiographic study found that the osteophyte size in the medial patellofemoral compartment correlated positively with the severity of narrowing in the medial tibiofemoral compartment [15]. In our model, the existence of osteophytes in the medial femoral trochlea can predict the radiographic progression of the medial tibiofemoral compartment in patients with knee OA. A previous study found that bone marrow lesions (BMLs) were associated with less tibial cartilage volume and that the severity of BMLs predicted greater cartilage loss over 2 years in patients with symptomatic knee OA [16]. Our study further identified BMLs in the medial trochlea of the femur as a predictor of radiographic progression of knee OA. An MRI study found a significant association between denuded areas of subchondral bone (dAB) in the femorotibial joint and increasing KLG. and with ipsicompartimental joint space narrowness. Eckstein et al. proposed a nomenclature for MRI-based measures of different parts of articular cartilage, which was adopted by OAI [17]. Using this terminology, we found the area ratio of subchondral bone denuded of cartilage (dAB %) in the external subregion of the central medial femur (ecMF) can predict the radiographic progression of knee OA.

In addition to MRI-based predictors, we found that lawn work also predicts radiographic progression. Physical activity may increase the risk of knee OA. A 48-month cohort study of subjects without radiographic knee OA or knee pain reported that people with jobs that required them to walk while handling certain materials were more likely to develop knee OA than people with jobs that mostly involved sitting [18].

There were three predictors based on pain-related queries in our predictive model of pain progression. A cross-sectional study found that unpredictable intermittent knee pain is likely associated with an unacceptable symptom state [19]. The WOMAC pain score at baseline was a negative predictor in the predictive model of pain progression, which might be due to tolerance of persistent chronic pain in patients with knee OA. Furthermore, our results showed that patients with increased WOMAC pain scores from baseline to 24 months were prone to pain progression over 24 to 48 months. Early treatment of knee pain and long-term control of knee pain by targeting the underlying mechanisms may be important in preventing pain progression in knee OA.

Back pain was a positive predictor of knee OA pain progression in our study. Low back pain was significantly associated with WOMAC knee pain score [20] and contributed to disability levels in individuals with knee OA [21]. Fu et al. found that subjects with spinal deformity (pelvic incidence - lumbar lordosis > 20°) showed higher pelvic tilt and knee flexion compared to normal subjects, indicating severe sagittal imbalance of the spine will lead to knee OA [22]. Enhancing core strengths may help alleviate back pain and prevent the worsening of knee OA pain.

NSAIDs are widely used to alleviate the symptoms of OA. However, how these drugs affect the progression of OA remains controversial. A meta-analysis showed that long-term use of NSAIDs, particularly nonselective drugs, accelerated structural progression [23]. Our results showed that the use of nonprescribed NSAIDs was associated with the progression of knee OA pain. Given that NSAID use is associated with a risk of OA progression, there is a need to evaluate alternative treatments to NSAIDs.

Using nine predictors, Dunn et al. developed a predictive model for end-stage knee OA progression [7]. In the present study, we compared the predictive efficiency between our models and the model constructed with the predictors reported by Dunn et al. via ROC curves and DCA. We found that our predictive model of radiographic progression has better predictive performance, which suggested that our model can better identify patients who are prone to structural progression in OA.

The FNIH OA Biomarkers Consortium investigated a set of biochemical biomarkers as predictors of OA progression to support new drug development, preventive medicine, and diagnostics [24]. Notably, some chemical biomarkers, such as urinary CTXII for radiographic progression and urinary CTX-1a for pain progression, were shown to be risk factors in the univariate analysis but were not included in the final multivariate model, probably due to their associations with other predictors.

There are also some limitations to our study. First, a majority of variables were initially excluded due to missing values. These variables may play an important role in the development of knee OA. Further analysis of these variables will help identify additional risk factors for OA progression. Second, the predictive models are highly dependent on MRI, and there are limited clinical indications for obtaining MRI in patients with early knee OA. However, in clinical trials of knee OA, MRI is recommended because of its sensitivity to structural changes. Third, although we cross-validated the results in the same cohort, it would be useful to reproduce them in another cohort to understand the consistency of the findings across cohorts, geographic regions, etc.

## Conclusions

In summary, our study provides models with good predictive value for radiographic progression and pain progression at 24 to 48 months in patients with knee OA. The models are useful not only for identifying patients at high risk of OA progression but also for selecting candidates for clinical trials to investigate treatments for OA.

### Electronic supplementary material

Below is the link to the electronic supplementary material.

Supplementary Material 1

## Data Availability

The datasets used and/or analyzed during the current study are available in the Osteoarthritis Initiative repository, publicly available at https://nda.nih.gov/oai [3].

## Abbreviations

OA: Osteoarthritis
OAI: Osteoarthritis Initiative
minJSW: Minimum joint space width
MFTC: Medial femorotibial compartment
WOMAC: Western Ontario and McMaster Universities Arthritis Index
VIF: Variance inflation factor
AIC: Akaike information criterion
AUC: Area under curve
ROC: Receiver operating characteristic
DCA: Decision curve analysis
SD: Standard deviations
BML: Bone marrow lesion
NSAIDs: Nonsteroidal anti-inflammatory drugs
KOOS: Knee Injury and Osteoarthritis Outcome Score
OARSI: Osteoarthritis Research Society International
